# Transcriptomic Analysis of the Anthocyanin Biosynthetic Pathway Reveals the Molecular Mechanism Associated with Purple Color Formation in Dendrobium Nestor

**DOI:** 10.3390/life11020113

**Published:** 2021-02-02

**Authors:** Xueqiang Cui, Jieling Deng, Changyan Huang, Xuan Tang, Xianmin Li, Xiuling Li, Jiashi Lu, Zibin Zhang

**Affiliations:** Flower Research Institute, Guangxi Academy of Agricultural Sciences, Nanning 530007, China; yncuixueqiang@gxaas.net (X.C.); jieling4149@163.com (J.D.); hcy23007@163.com (C.H.); tangxuan0606@163.com (X.T.); lixm7406@126.com (X.L.); congzxiao@163.com (X.L.)

**Keywords:** orchid, pigmentation, anthocyanin biosynthesis, gene coexpression, transcription factors

## Abstract

Dendrobium nestor is a famous orchid species in the *Orchidaceae* family. There is a diversity of flower colorations in the *Dendrobium* species, but knowledge of the genes involved and molecular mechanism underlying the flower color formation in D. nestor is less studied. Therefore, we performed transcriptome profiling using Illumina sequencing to facilitate thorough studies of the purple color formation in petal samples collected at three developmental stages, namely—flower bud stage (F), half bloom stage (H), and full bloom stage (B) in D. nestor. In addition, we identified key genes and their biosynthetic pathways as well as the transcription factors (TFs) associated with purple flower color formation. We found that the phenylpropanoid–flavonoid–anthocyanin biosynthesis genes such as phenylalanine ammonia lyase, chalcone synthase, anthocyanidin synthase, and UDP-flavonoid glucosyl transferase, were largely up-regulated in the H and B samples as compared to the F samples. This upregulation might partly account for the accumulation of anthocyanins, which confer the purple coloration in these samples. We further identified several differentially expressed genes related to phytohormones such as auxin, ethylene, cytokinins, salicylic acid, brassinosteroid, and abscisic acid, as well as TFs such as MYB and bHLH, which might play important roles in color formation in D. nestor flower. Sturdy upregulation of anthocyanin biosynthetic structural genes might be a potential regulatory mechanism in purple color formation in D. nestor flowers. Several TFs were predicted to regulate the anthocyanin genes through a K-mean clustering analysis. Our study provides valuable resource for future studies to expand our understanding of flower color development mechanisms in D. nestor.

## 1. Introduction

The Orchidaceae family comprises orchids from 736 genera and represents the second largest family of flowering plants [1,2]. *Dendrobium* species represent important orchids that are either epiphytic or lithophytic and have frequent buds [1,3]. The species is distributed and cultivated in tropical Asia, Australasia, and Australia [4]. More than 74 species and two widely cultivated genotypes of this genus were reported in China, some of which are famed for their flowers during Father’s Day in Asia [4]. Dendrobium nestor is derived from a cross between *Dendrobium parishii* x *D. anosmum*, and its flowers present color diversity, spanning from white, red, to purple [5]. The color variations in the D. nestor flower contribute to the release of fragrance as attractants for pollinators [6]. Color variations in orchids account for their aesthetic and commercial values in the ornamental industry [2]. The purple color variant in D. nestor substantially accounts for the visual appeal and market value of the flowers. *Dendrobium* orchids are also highly employed for their medicinal values. Several bioactive compounds and metabolites housed in these species are useful for human health, including nourishing the kidney, enhancing the body’s immunity, and resisting cancer [4].

Anthocyanins are part of flavonoids, the phenolic compounds that determine flower, fruit, and seed color in several plant species [7,8,9,10]. They are key members of the phenylpropanoid biosynthetic pathway [11,12] and are water-soluble, synthesized in cytosol, stored in the vacuole, and encode for the blue, purple, red, or white colors of flowers and fruits [13]. Three aromatic rings characterize these compounds and could be substituted by acyl, hydroxyl, methyl, and sugar, depending on the plant species [7]. Biosynthesis of anthocyanins are controlled by structural and regulatory genes involved in the formation of enzymes, and regulation of expression of specific enzymes [10,14]. In petunia flowers, differential expression of anthocyanin structural genes result in spot color formation [15,16].

Major genes participating in the anthocyanin biosynthetic pathway encode flavanone 3-hydroxylase, flavonoid 3′-hydroxylase, dihydroflavonol 4-reductase, chalcone synthase, chalcone isomerase, cinnamate 4-hydroxylase, 4-coumaroyl CoA ligase, glutathione S-transferase, leucoanthocyanidin dioxygenase, phenylalanine ammonia-lyase, and UDP-flavonoid glucosyl transferase [17]. Transcription factors (TFs) control gene expression in numerous biological processes in plants [5]. The regulatory mechanisms of a number of genes involved in the anthocyanin biosynthetic pathway are well characterized in plants [6,18,19,20,21]. For instance, a flower-specific MYB protein activation of genes involved in phenylpropanoid biosynthesis was reported in *Antirrhinum* [22,23]. In Arabidopsis, *PAP1*/*MYB75, PAP2/MYB90, MYB113*, and *MYB114* control anthocyanin biosynthesis [24,25]. Likewise, it was demonstrated that *TTG1* (WD40), *GL3/EGL3/TT8* (bHLH), and *PAP1/PAP2/MYB113/MYB114* (MYB) for a WBM complex that controls anthocyanin biosynthesis [26,27]. The implication of WRKY TFs in the co-regulation of anthocyanin via the MBW complex were documented in Arabidopsis and Petunia [18,28]. Additionally, the bZIP TF family members are potential regulators of the anthocyanin pathway in apple [29].

Although extensive knowledge are available in different plant species concerning the molecular mechanisms of color formation, species-specific distinctiveness in the transcriptional regulation of color formation in plants was reported. Currently, the role of the anthocyanin biosynthetic pathway in petal/flower color formation in D. nestor remains unclear. Identifying the key genes related to color formation in the D. nestor flower would facilitate the manipulation of the related pathways to develop new cultivars with specific flower colors. In recent years, transcriptome sequencing (RNA-seq) was used as a rapid technique to uncover differentially expressed genes, TFs, and biosynthesis pathways related to specific traits in plants [13,30,31]. Transcriptome sequencing of the D. nestor petals/flowers would provide useful insights into the genetics of purple color formation. To the best of our knowledge, there is limited study on flower coloration in D. nestor, and the molecular mechanisms of the flower coloration and regulation remains obscure. In this study, we performed transcriptome sequencing on the petals of blooming flowers of D. nestor at different periods to discover key candidate genes of the anthocyanin biosynthetic pathway underlying the purple color formation. Our results serve as a reference for understanding the regulation of key genes and transcription processes in purple color formation in the flowers of this aesthetically important orchid.

## 2. Material and Methods

### 2.1. Plant Growth and Sample Preparation 

**Figure 1:**
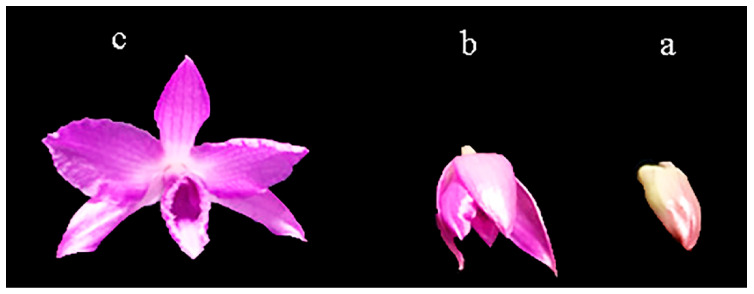
Petals of *Dendrobium nestor* at different stages of development. (**a**) Flower bud stage (F), (**b**) half blooming stage (H), and (**c**) full blooming stage (B).

D. nestor produces a large number of flowers, rich floral fragrance, gorgeous colors, and is highly appreciated by consumers. The genetic material used in the present study (D. nestor) was derived from a cross between *Dendrobium parishii × D. anosmum.* The color of the D. nestor flower at the full blooming stage is purplish. The D. nestor plants were cultivated in a greenhouse at the Flower Research Institute, Guangxi Academy of Agricultural Sciences, Nanning, China. A total of 12 petals were collected from 12 plants at three different stages of flower development, i.e., the flower bud stage (F, 5 mm long); half blooming stage (H, 10 mm long) and blooming stage (B, ˃11 mm long), and mixed to constitute a biological replicate (Figure 1). Sampling was conducted in three biological replicates at each flower developmental stage. Samples were immediately lyophilized in liquid nitrogen and stored at –80 °C and used in future experiments.

### 2.2. RNA Extraction, Sequencing Libraries, and RNA-Seq 

Total RNA was extracted from each sample (3 samples × 3 biological repeats) of D. nestor using Trizol reagents (Invitrogen, Carlsbad, CA, USA). The transcriptome library was constructed using the Illumina TruSeq Stranded total RNA with Ribo-Zero Globin (Illumina, San Diego, CA, USA), according to the manufacturer’s instructions. All cDNA libraries were subjected to high throughput sequencing using the Illumina HiSeq™ 4000 platform at Biomarker Technologies (Beijing, China). The raw sequencing data were cleaned and assembled using the Trinity software [32]. Gene expressions were normalized to the fragments per kilobase of transcript per million (FPKM) by HTSeq [33]. The expressions of genes were heatmapped and visualized using the Toolkit for Biologists, integrating various biological data handling tools [34].

### 2.3. Sequenced Data Filtering, Assembly, and Functional Annotation

The raw sequenced data with low-quality linker contaminants and reads with too high and unknown base contents were filtered using the FastQC software version 0.11.9 [35] to ensure reliability of results. The clean reads were used as candidates for the transcriptome assembly. The polymerase chain reaction (PCR) duplicates were removed with the Trinity program (version 2.8.4) [32]. The assembled transcripts were then clustered and a cluster deduplication was performed to obtain the final unigenes. The unigenes were named according to the cluster number, followed by its serial order. The assembled unigenes were annotated using the Clusters of Orthologous Groups of proteins (COG) [36], Gene Ontology (GO) (https://www.geneontology.org [37]), Kyoto Encyclopedia of Genes and Genomes (KEGG) [38], Pfam [39], translated European Molecular Biology Laboratory (TrEMBL; [40]), the NCBI non-redundant protein sequence database (Nr) (https://ftp.ncbi.nih.gov/blast/db/FASTA/nr.gz; [41]), and Eukaryotic Clusters of the Orthologous Groups (KOG, https://www.ncbi.nlm.nih.gov/COG/new/shokog.cgi). Principal Components Analysis (PCA) and Pearson correlation were performed on the basis of gene expression profiles obtained from FPKM, among the nine samples, in R [42]. 

### 2.4. Detection of Differentially Expressed Genes and Functional Enrichment Analysis 

The uniquely mapped reads were retained for the unigene expression analysis. Differential expression analysis between treatments was performed based on FPKM, using the *DESeq* R package (1.10.1) with the Benjamini and Hochberg’s correction [43]. The unigenes with a false discovery rate (FDR) of ≤ 0.001 and a fold change of ≥ 2.0 or ≤ 0.5 in any pairwise comparison (F_vs_H, F_vs_B and H_vs_B) were considered as differentially expressed unigenes, which were designated as differentially expressed genes (DEGs). The DEGs were clustered by the Short Time-series Expression Miner (STEM) clustering analysis (STEM software), by setting 30 as the maximum number of model profiles, and 1 as the maximum unit change in model profiles between time-points [44]. The Kyoto Encyclopedia of Genes and Genomes (KEGG) database (https://www.genome.jp/kegg) was used for the functional annotation of specific purple color-conferring DEGs with the BLAST software [45] to identify anthocyanin biosynthetic pathways among the samples, in pairwise comparisons with a minimum threshold of statistical significance at *p* < 0.05.

### 2.5. Identification of Transcription Factors and Simple Sequence Repeats 

To predict transcription factors (TFs) involved in purple color formation in *D. nestor,* we utilized the getorf database (mini-size 150) to find the open reading frame (ORF) [46] and then used the HMM search database (version 3.0) to align the ORFs to the TF protein domain [47]. The aligned sequences were described according to the TF families available on the PlantTF database version 3.0 [48]. The online Perl script program, MIcroSAtellite identification tool (MISA; http://pgrc.ipk-gatersleben.de/misa/) was employed to identify SSRs in *D. nestor* using the default settings. Primer3 software (ver 2.2.2) was used to design primers for the detected SSRs [49].

### 2.6. Real-Time Quantitative Polymerase Chain Reaction Analysis 

Primers were designed using the Primer 4.0 tool (Additional File 8: Table S6). The qRT-PCR was performed using the LightCycler 480 with Unique AptamerTM qPCR SYBR® Green Master Mix (Novogene Technology Co. Ltd., Beijing, China), according to the manufacturer’s instructions. The gene *Actin* (JX294908) was used as the housekeeping gene. All reactions were performed in biological triplicates and technical triplicates. Relative expression values were computed using the 2^−∆∆^Ct method [50]. Student *t*-test was used to separate the means, thus, the difference was considered to be statistically significant at *p* < 0.05.

## 3. Results

### 3.1. Sequencing Summary, Assembly, and Unigene Annotation

Flower samples collected in triplicates at three stages of development (F = flower bud stage; H = half blooming stage; and B = full blooming stage) from D. nestor plants were used for RNA-seq (Figure 1). The purple coloration increased gradually from F to B (Figure 1). The RNA-seq was conducted on nine cDNA libraries (3 samples × 3 repeats) and generated, on average, 50,047,108 (F), 48,759,280 (H), and 51,171,054 (B) raw reads, (Table 1). After filtering the contaminants, the average clean reads ranged from 46,903,317 (95.05% of the raw reads) to 49,217,131 (96.28% of the raw reads) corresponding to 7.03–7.38 Gb clean reads. Each sample had a Q30 and GC content above 93% and 46%, respectively (Table 1).

**Figure 2:**
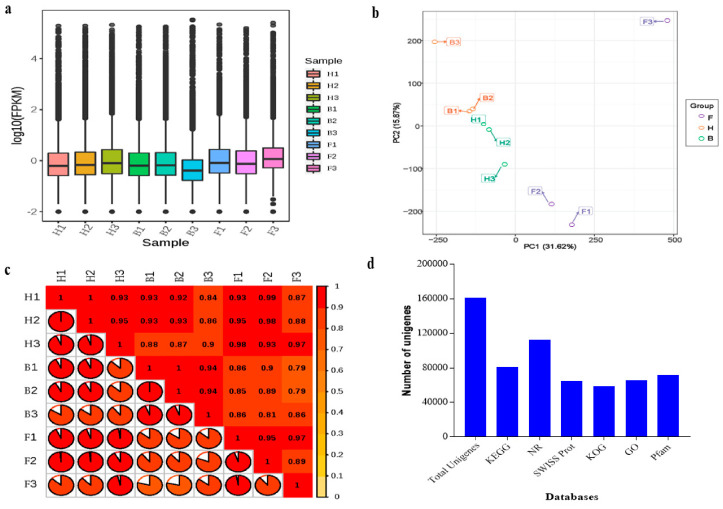
Transcripts and unigenes obtained from the petals of *D. nestor* at three stages of development (F, H, and B). (**a**) Overall distribution of sample gene expression based on fragments per kilobase of transcript per million fragments mapped (FPKM). (**b**) Principal component analysis based on FPKM. (**c**) Pearson correlation between three replicates of the three samples. (**d**) Number of unigenes in the pairwise groups (F_vs_H, F_vs_B, and H_vs_B) annotated to different databases. Where F = flower bud stage; H = half blooming stage; and B = full blooming stage.

The clean libraries were assembled with the program Trinity [32]. The assembler generated a total of 220,258 transcripts, out of which 59,986 (27.23%) had a length of 200–300 bp (Additional File 1: Figure S1). Subsequently, a cluster deduplication was performed to obtain the final unigene for analyses. As a result, a total of 161,228 unigenes were obtained, of which 26,998 (16.75%) had a mean length ≥2000 bp (Additional File 1: Figure S1). The overall distribution of the gene expression was based on the fragments per kilobase of transcript per million fragments mapped (FPKM) procedure (Figure 2a). The use of relative unigene expression obtained from FPKM for principal component analysis (PCA) showed 47.49% variability among the three samples (F, H, and B) (Figure 2b). However, biological replicates had a very strong correlation co-efficient (r ≥ 0.79), as evidenced in Figure 2c. Overall, we detected 46,770, 45,739, and 38,601 expressed unigenes in F, H, and B samples, respectively.

From above, 112,581 unigenes (representing 69.83%) were successfully annotated to at least one of the six public gene functional annotation databases, including KEGG, Nr, SWISS Prot, KOG, GO, and Pfam via BLAST. Figure 2d gives annotation across the six databases. The high-quality reads, variability among the sample groups, and similarity within biological replicates confirmed the reliability of our data for further analyses.

**Figure 3:**
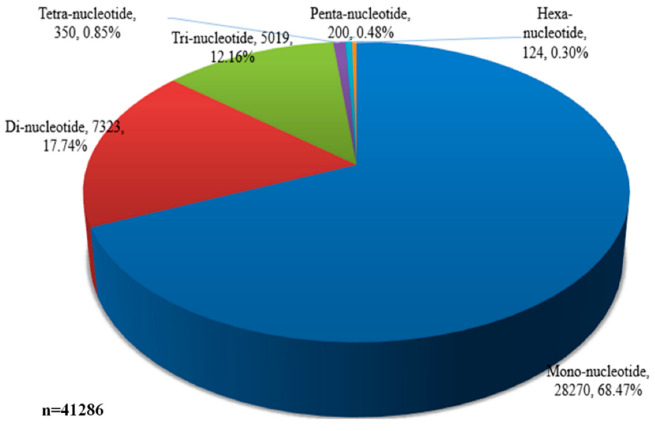
Type of simple sequence repeats detected in the transcriptome; n = total number of markers.

Transcriptome assembly of D. nestor provides the opportunity to detect simple sequence repeats (SSR), which could be useful for a variety of identification and molecular assisted breeding. In total, we identified 41,286 SSR markers, dominated by mono-nucleotide (68.47%), di-nucleotide (17.47%), and tri-nucleotide (12.16%) SSR types (Additional file 2: Table S1, Figure 3).

### 3.2. Differentially Expressed Genes and Functional Enrichment Analyses

**Figure 4:**
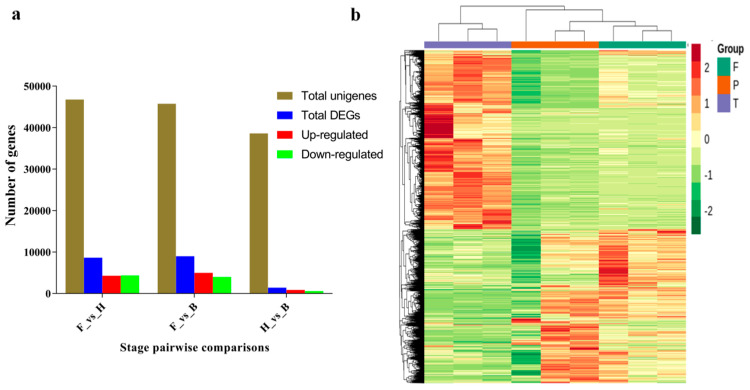
Number of differentially expressed genes (DEGs) relative to the total number of unigenes obtained from the petals of D. nestor in pairwise groups (F_vs_H, F_vs_B, and H_vs_B). (**a**) Total number of unigenes, DEGs, and their extent of regulation (either up- or down-regulated). (**b**) Heatmap obtained based on FPKM of DEGs. Where F = flower bud stage; H = half blooming stage; and B = full blooming stage.

Differentially expressed genes (DEGs) were selected based on a threshold of log2 fold change (log_2_FC) ≥ 1 and false discovery rate (FDR) ≤ 0.05. We obtained a total of 8627, 8993, and 1418 DEGs in F_vs_H, F_vs_B, and H_vs_B, respectively, from 46,770, 45,739, and 38,601 unigenes (Figure 4a; Additional file 3: Table S2a–c). Based on the DEGs, two main clusters of flower samples were obtained (Figure 4b). Cluster one mainly comprised F samples, whereas cluster two consisted of H and B samples. This implied that H and B samples had some similarity in transcriptional activity. This similarity might be due to relatively similar anthocyanin synthesis and accumulation at the half blooming stage (H) and full blooming stage (B). The ten DEGs selected for qRT-PCR to validate our RNA-seq data had similar relative expression pattern with the RNA-seq data (Additional file 4: Figure S2).

The DEGs obtained in each pairwise group were employed for the KEGG pathway enrichment analysis. From the KEGG pathway enrichment analysis based on the *p*-value of significance (Additional File 5: Table S3), the most significant pathways were phenylpropanoid biosynthesis, biosynthesis of secondary metabolites, plant hormone signal transduction, metabolic pathways, carotenoid biosynthesis, and the circadian rhythm of the plant. Given that phenylpropanoid (particularly anthocyanin biosynthesis pathway) and plant hormone signal transduction are well known to modulate color formation in plants, we focused on these as the candidate pathways to elucidate their involvement in petal/flower color formation in D. nestor.

### 3.3. DEGs Involved in Anthocyanins Biosynthesis Pathway

**Figure 5:**
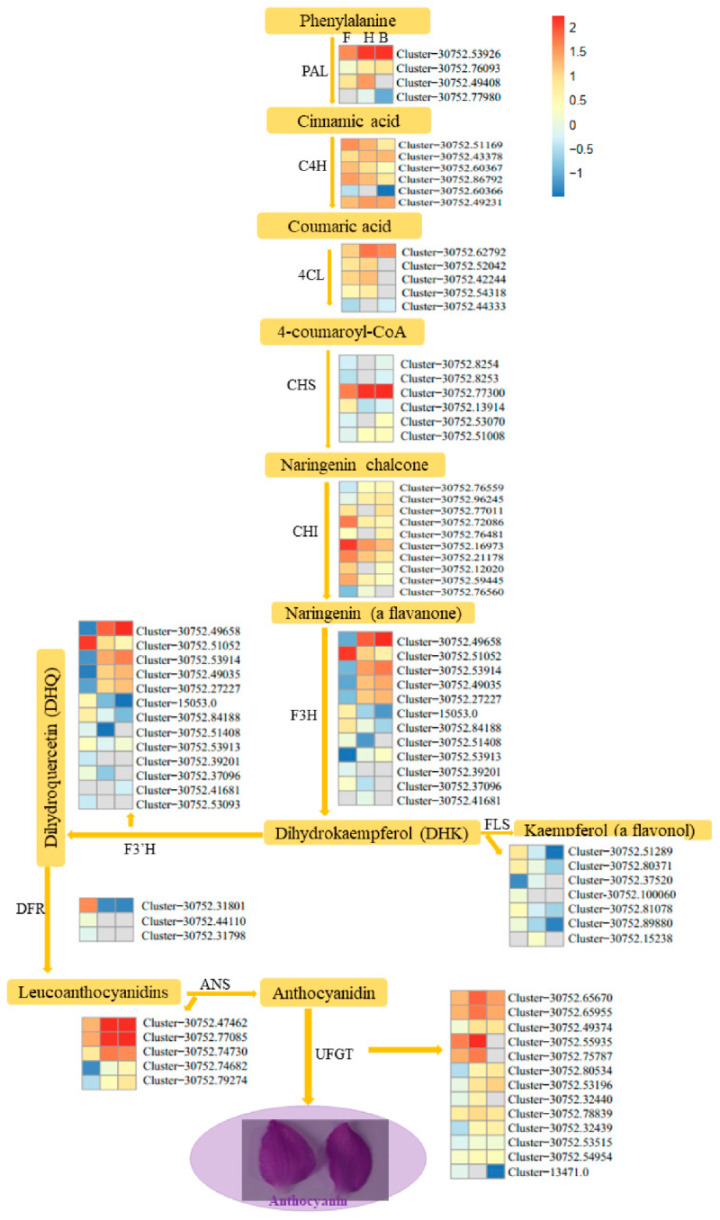
A schema of the anthocyanin biosynthesis pathway with the differentially expressed genes (DEGs) in the F, H, and B samples and their related enzymes. The log10 transformed fragments per kilobase of transcript per million fragments mapped the values of DEGs linked to enzymes were used to draw to the heatmap with the pheatmap package in R [53]. The enzymes include phenylalanine ammonia lyase (PAL), 4-coumarateCoA ligase (4CL), chalcone synthase (CHS), chalcone isomerase (CHI), flavone 3-hydroxylase (F3H), flavonoid 3′-hydroxylase (F3′H), flavonol synthase (FLS), dihydroflavonol reductase (DFR), and UDP-flavonoid glucosyl transferase (UFGT). F refers to the flower bud stage; H refers to the half blooming stage; B refers to the full blooming stage. The color gradient comprisess chocolate, ghostwhite, and blue, representing the upregulated, not regulated, and downregulated genes, respectively.

The key determinant of flower color is the composition and concentrations of anthocyanins, carotenoids, and betalains as key pigments [25]. Anthocyanins are responsible for orange, pink, red, purple, blue, and blue-black flower colors [25,51]. Anthocyanins are flavonoids, which are a major offshoot of the highly branched phenylpropanoid pathway with several enzymes involved [52]. The main amino acid precursor for phenylpropanoids is phenylalanine (phenylalanine ammonia lyase, PAL). In our present study, four key genes (Cluster-30752.53926, Cluster-30752.76093, Cluster-30752.77980, and Cluster-30752.49408) linked to PAL were highly up-regulated in the H and B samples, as compared to the F sample, with the exception of Cluster-30752.49408, which had no expression in the P sample (Figure 5). Similarly, two out of six genes (Cluster-30752.43378 and Cluster-30752.49231) linked to cinnamate 4-hyroxylase (C4H) in converting cinnamic acid to coumaric acid were highly expressed in the H and B samples relative to the F sample. In addition, enzymes: 4-coumarateCoA ligase (4CL), chalcone synthase (CHS), chalcone isomerase (CHI), flavone 3-hydroxylase (F3H), flavonoid 3′-hydroxylase (F3′H) and flavonol synthase (FLS) were associated with 5, 6, 10, 12, 13, and 7 DEGs, respectively. Out of these, most were highly expressed in the H and B samples, as compared to the F sample (Figure 5). Surprisingly, three genes (Cluster-30752.31801, Cluster-30752.44110, and Cluster-30752.31798) linked to dihydroflavonol reductase (DFR) were more highly expressed in the F sample than either in the H or B sample; this enzyme converts Dihydroquercetin (DHQ) to Leucoanthocyanidins (Figure 5). Anthocyanidin synthase (ANS) converts the Leucoanthocyanidins to anthocyanidin and we identified five DEGs including, Cluster-30752.47462, Cluster-30752.77085, Cluster-30752.74730, Cluster-30752.74682, and Cluster-30752.79274. These genes were highly expressed in the H and B samples relative to the H sample (Figure 5). Thirteen DEGs encoding the UDP-flavonoid glucosyl transferase (UFGT) were more highly expressed in the H or B samples than in the F sample (Figure 5). These results showed that globally, most genes involved in the early and late steps of the anthocyanin biosynthetic pathway were strongly upregulated over the flower development stages in D. nestor and might be critical for the purple color formation. 

### 3.4. Plant Hormone Signal Transduction

**Figure 6:**
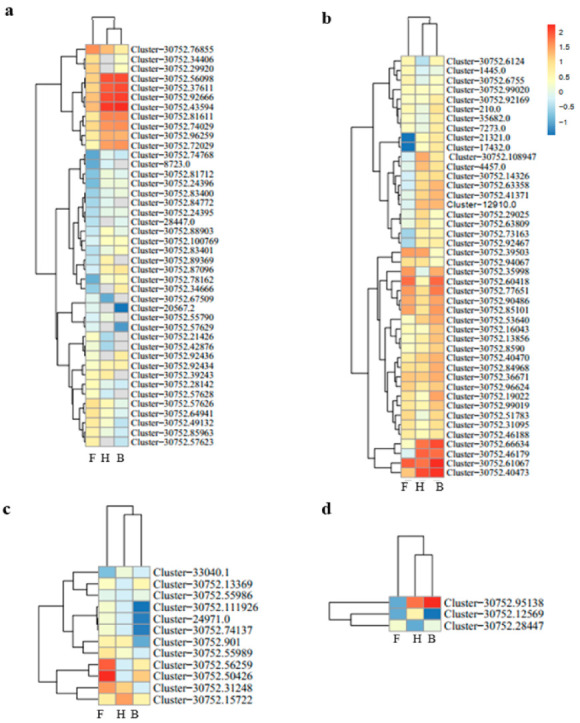

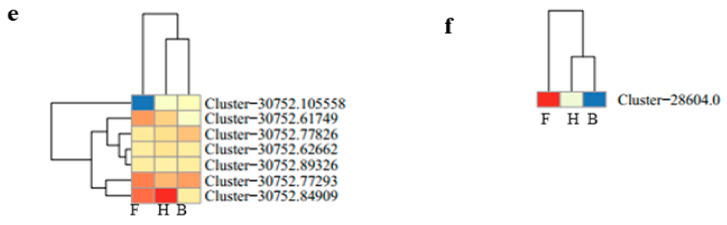
Expression profile based on standardized (log10 transformed) fragments per kilobase of transcript per million fragments mapped (FPKM) values of differentially expressed genes (DEGs) related to phytohormones with the Euclidean distance method implemented with the *pheatmap* package in R [53]. (**a**) Auxin, (**b**) Ethylene, (**c**) Brassinosteroid, (**d**) Abscisic acid, (**e**) Cytokinin, and (**f**) Salicylic acid. Where F = flower bud stage; H = half blooming stage; and B = full blooming stage. The color gradient comprises chocolate, ghostwhite, and blue, representing upregulated, not regulated, and downregulated genes, respectively.

Beside environmental stimuli such as light and temperature, phytohormones (auxin, ethylene, cytokinins, salicylic acid, brassinosteroid, and abscisic acid (ABA)) significantly influence anthocyanin biosynthesis [54,55,56,57,58,59]. Most DEGs involved in auxin and ethylene were largely upregulated in the H and B samples relative to the F sample (Figure 6a,b). On the contrary, DEGs related to brassinosteroid, ABA, cytokinins, and salicylic acid were mostly repressed in the H and B samples, as compared to the F sample (Figure 6c–f). All phytohormones related to DEGs were clustered under the three samples (F, H, and B) into two sub-clusters. With the exception of cytokinins, all phytohormones clustered with the H and B samples into one sub-cluster, while the F sample was in another sub-cluster. This supports the trend of increasing purpleness, as observed in Figure 1. These trends showed various modulating roles of different phytohormones in color formation in D. nestor. In addition, these signify the important roles of plant hormone signal transduction in modulating petal color development in D. nestor. 

### 3.5. Identification of Transcription Factors Regulating Color Formation in the Petals/Flowers of D. nestor 

Transcription factors (TFs) are the primary regulators of gene expression [60], of which many were predicted to modulate anthocyanin accumulation and biosynthesis in flowering plants. The sequences of DEGs obtained in each pairwise group were submitted to the plant transcription factor database to identify DEGs encoding for TFs. A total of 601 (F_vs_H), 583 (F_vs_B), and 116 (H_vs_B) DEGs encoding TF were detected, with varied degree of regulation (Additional File 6: Table S4a–c). The AP2/ERF, bHLH, bZIP, C2H2, C3H, HB, MADS-MIKC/MADS-M-type, NAC, and WRKY TFs were largely upregulated in F_vs_H and F_vs_B but were downregulated in H_vs_B (Table 2). However, C2C2, MYB, and Tify TFs were mostly downregulated in the three pairwise groups (Table 2). 

**Figure 7:**
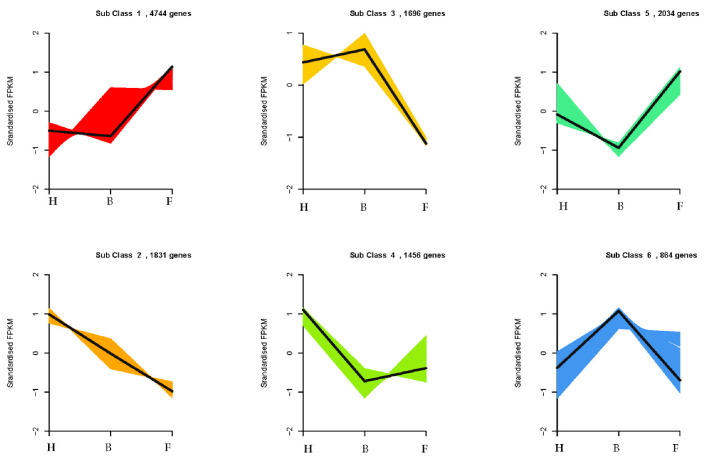
K-means clustering of differentially expressed genes based on standardized (log10 transformed) fragments per kilobase of transcript per million fragments mapped values of three samples of D. nestor flowers (F, H, and B). The numbers of genes clustered in each subclass are given above each Figure. Where F = flower bud stage; H = half blooming stage; and B = full blooming stage.

In addition, a K-means clustering analysis based on the FPKM of 12,625 unique genes among the samples (F, H, and B) was conducted by following the procedure outlined by [61] to identify co-expressed modules. Importantly, the TFs co-expressed with the anthocyanin biosynthetic genes could play important roles in modulating purple coloration in the petal/flower of D. nestor. In all, six clusters of genes were detected (Figure 7). Most genes involved in the anthocyanin biosynthesis pathway were grouped in Cluster 1 with several TF family members, including MYB, bHLH, WRKY, and AP2/ERF (Additional file 7: Table S5). For instance, *Cluster-30752.54898*, *Cluster-30752.54899*, and *Cluster-30752.54900* in Cluster 1 have bHLH-MYC and R2R3-MYB TFs N-terminal domain. 

**Figure 8:**
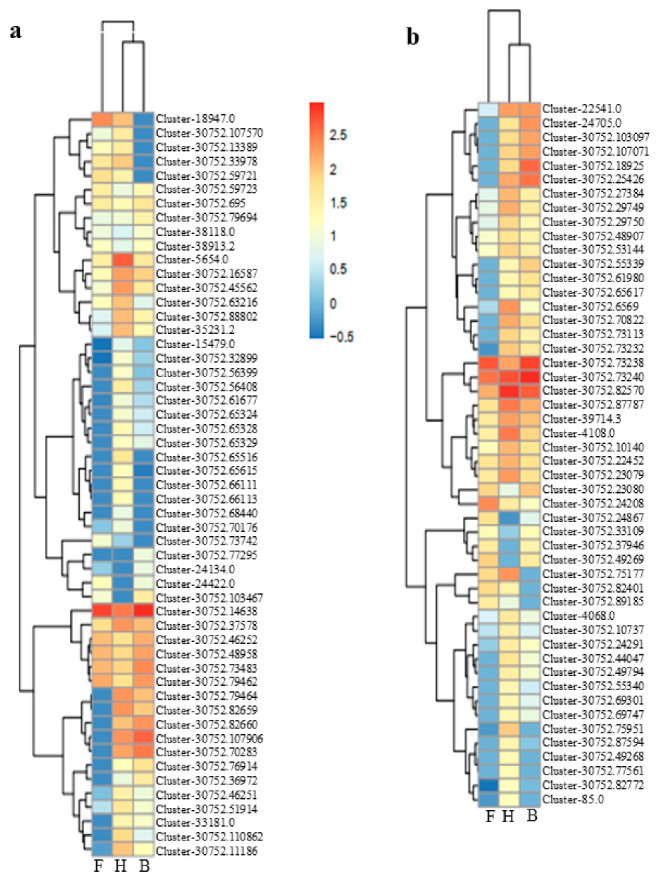
Expression profile of (**a**) MYB and (**b**) bHLH transcription factor families with differentially expressed genes based on standardized (log10 transformed) fragments per kilobase of transcript per million fragments mapped values of three samples of D. nestor flowers (F, H, and B ) constructed with the *pheatmap* package in R [53]. Where F = flower bud stage; H = half blooming stage; and B = full blooming stage. The color gradient comprises chocolate, ghostwhite, and blue, representing upregulated, not regulated, and downregulated genes, respectively.

Genes involved in the anthocyanin pathways are differentially modulated in monocots and dicots by MYB, bHLH, and WD40 TFs [62,63]. Hence, we studied MYB and bHLH TFs further to deepen our understanding of their involvement in modulating purple flower color formation at three different stages in *D nestor*. A total of 53 and 50 genes with MYB and bHLH were profiled for their expressions among the F, H, and B samples, based on standardized FPKM values (Figure 8a,b). Most of these genes were largely upregulated (58.49% and 62%) in the F sample, as compared to the H and B samples. Five MYB TFs: *Cluster-30752.45562*, *Cluster-30752.14638*, *Cluster-30752.37578*, *Cluster-30752.48958*, and *Cluster-30752.73483* and another five bHLH TFs: *Cluster-30752.24208*, *Cluster-30752.33109*, *Cluster-30752.37946*, *Cluster-30752.89185*, and *Cluster-4068.0* potentially modulated increasing purple coloration from the F to the B sample. However, this needed further functional validation to ascertain their specific roles in color formation.

## 4. Discussion

Flowers offer visual appeal, and the commercial value of ornamental plants is markedly determined by petal color. Previous studies on floral coloration revealed species-specific distinctiveness in pigment regulation [25,64,65,66,67]. Additionally, the variations in floral coloration emanates from different processes, such as competition among pathways, expression patterns of structural genes involved in pigment formation, and mutations in structural or regulatory genes [24,68]. Thus, species-specific studies of flower color formation and the elucidation of their regulatory mechanisms are essential to deepen our understanding of flower color development in orchids. In plant breeding and horticulture, transcriptome sequencing is highly employed for predicting novel genes, gene function, and genome evolution [11]. Here, we performed whole-transcriptome sequencing to study the mechanism of purple color development in D. nestor petals, which is the first report, as compared to other Dendrobium species [13,69,70,71,72]. H and B samples might have relatively similar anthocyanins accumulation and synthesis, hence the high similarity as evident in Figure 1a,b and Figure 4b. We also reported the first ever SSR markers useful for future molecular breeding efforts in D. nestor (Figure 3, Additional file 2: Table S1). 

### 4.1. Pathways Involved in Purple Color Formation in Petals of D. nestor at Different Stages of Development

Flowering plants exhibit a wide variation in their flora, foliage, and fruit colors, as a result of genetic factors and variations in environments. Flower color formation in orchids are controlled largely by anthocyanins, carotenoids, and betalains [25]. In this present study, petals/flowers of D. nestor were sampled at different developmental stages to conduct transcriptome sequencing. From the KEGG pathway enrichment analyses, the most prominent pathways were phenylpropanoid–flavonoid–anthocyanin biosynthesis, biosynthesis of secondary metabolites, plant hormone signal transduction, metabolic pathways, carotenoid biosynthesis, and the circadian rhythm plant biosynthesis pathway (Figure 4a–c; Additional file 5: Table S3). Ma et al. [73] and Luo et al. [74] reported these to be the predominant pathways in color formation in *Vitus vinifera* and *Punica granatum*, which was consistent with the results of this study.

Anthocyanins are flavonoids, which are a major offshoot of the highly branched phenylpropanoid pathway, with several prominent genes including, *PAL*, *C4H*, *4CL, CHS, CHI, F3H, F3′H, FLS, DFR, ANS*, and *UFGT* [52]. Our results revealed most of the *PAL*, *CHS*, *ANS,* and *UFGT* genes involved in anthocyanins biosynthesis were up-regulated and increased in abundance in the H and B samples, as compared to the F sample (Figure 5). These genes might have contributed to the increasing purple coloration from the F sample to the H and B samples, as evidenced in Figure 1. The *CHS* provides the precursor for 4-coumaroyl-CoA to naringenin chalcone in the flavonoid biosynthesis pathway. The CHS encoded genes (*Cluster-30752.8254, Cluster-30752.8253, Cluster-30752.53070*, *Cluster-30752.77300*, and *Cluster-30752.51008*), which were highly expressed in the B sample, relative to the F and H samples. However, the latter two genes were more highly expressed in the H sample than F sample (Figure 5). These genes are involved in the key regulatory step during the synthesis of flavonoids, which is a major pigment in many flowers, leaves, and fruits [75,76]. The Naringenin is converted into several anthocyanin-related substances like dihydrokaempferol, by the action of the F3H encoded genes, which were mostly expressed the most in the B samples than either the F or H samples (Figure 5). Earlier study identified *PaCHS* as one of the modulators of purple coloration in *Phalaenopsis amabilis* [77]. *SlCHS* contributes to red coloration in tomato [78], while *CaCHS* regulates anthocyanin-pigmented fruits in *Capsicum annuum* [79]. The *ANS* converts the leucoanthocyanidins to anthocyanidins (Figure 5). It was previously reported that *CaANS* increases from the young stage and reaches maximum at the late unripe stage, prior to ripening in *C. annuum* [79]. A similar trend was observed for the five *ANS* genes (*Cluster-30752.47462*, *Cluster-30752.77085*, *Cluster-30752.74730*, *Cluster-30752.74682*, and *Cluster-30752.79274*) in the three samples of flowers (Figure 5). Similarly, expression levels of genes encoding ANS were upregulated and the abundance of their corresponding proteins also increased during fruit color development [74]. Another prominent gene, *UFGT*, which glycolyzes anthocyanidin into anthocyanin [80], gene13307, and gene43584 were down-regulated in the low anthocyanin *Yunyan 87* mutant of tobacco [81]. In our study, ten *UFGT* genes (*Cluster-30752.65670*, *Cluster-30752.65955*, *Cluster-30752.49374*, *Cluster-30752.55935*, *Cluster-30752.75787*, *Cluster-30752.80534*, *Cluster-30752.53196*, *Cluster-30752.32440*, *Cluster-30752.78839*, *Cluster-30752.32439*, *Cluster-30752.53515*, and *Cluster-30752.54954*) were highly expressed in the H and the B sample, as compared to that of the F sample (Figure 5).

Anthocyanin biosynthesis pathway is modulated by a number of phytohormones [54,55,56,57,58,59]. He et al. (2020) highlighted that endogenous auxin (indole acetic acid) and ABA play diverse and specific roles in *D. officinale* color development. In our study, 25 of the 43 genes involved in auxin signaling pathway were more highly expressed in either the H or the B samples than in the F samples (Figure 6a). This is not surprising as auxins (naphthalene acetic acid) or 2,4-dichlorophenoxyacetic acid modulates secondary metabolic pathways, including those related to phenylpropanoid, flavonoid, and anthocyanin metabolism [82,83]. Additionally, most genes involved in ethylene signaling were upregulated in the largely anthocyanin-accumulated samples (H or B) (Figure 6b). This contradicted reports by Jeong et al. [55] that ethylene signaling negatively regulates anthocyanin accumulation. Our results provide evidence of phenylpropanoid–flavonoid–anthocyanin biosynthesis, and plant hormone signal transduction pathways modulating flower coloration in D. nestor. Although in this study we provided a good insight into the expression patterns of the anthocyanin-biosynthesis-pathway-related structural genes, we did not perform any analytical analysis to identify the key pigments conferring the purple coloration in D. nestor petals. Hence, we are designing a future study that would clarify this to consolidate the molecular data reported in the present work.

### 4.2. Transcriptional Regulation of Purple Color Formation in D. nestor 

Gene expression is a complex process and involves the coordination of multi-dynamic events, which are subject to multi-level regulation [84], including transcriptional, post-transcriptional, translational, and post-translational events. Transcription factors (TF) perform two important roles in flowering plants—they recognize and bind to short, specific sequences of DNA within the regulatory region. Second, they recruit or bind to proteins that participate in transcriptional regulation [85]. The most abundant TFs predicted in this study were AP2/ERF, AUX/IAA, bHLH, bZIP, MYB, NAC, Tify, WRKY, and other families (Table 2; Additional File 6: Table S4a–c). The class of TFs identified were previously implicated in the modulation of color formation in petals/flowers of orchids, such as roses [29,30,31]. For example, five bHLH-MYC and R2R3-MYB were more highly expressed in the H and B samples than in the T samples (Additional File 6: Table S4b,c). These indicate that some bHLH and MYB TFs were positive regulators of anthocyanins biosynthesis in D. nestor. These were in consonance with earlier reports that anthocyanins biosynthesis is transcriptionally regulated by the MYB–bHLH–WD40 complex [22,86,87]. In apple, *MdMYB1* and its alleles, MdMYB10 and MdMYBA, act as positive modulators of anthocyanin biosynthesis, by activating the expressions of *MdDFR* and *MdUF3GT* [88,89,90]. On the contrary, downregulation of MdMYB1 inhibits anthocyanin accumulation mediated by ethylene, abscisic acid (ABA), wounding, drought, and different light intensities [91,92,93,94]. Another abundant TF family, WRKY, was highly expressed in the H and B samples, as compared to the F samples, indicating that WRKY play a significant role in modulating anthocyanins biosynthesis. Recent studies on proanthocyanin and anthocyanin biosynthesis pathways in Arabidopsis and Petunia revealed that WRKY regulates the color development in concert with the MYB–bHLH–WD40 complex [95,96]. 

Similarly, most genes with NAC TFs increased in expression from the F, to the H and B samples. In peach (*Prunus persicae*), *PpNAC1* activates the transcription of *PpMYB10.1*, leading to anthocyanin pigmentation in tobacco and apple [97,98]. In contrast, *PpNAC1* was silenced resulting in a reduction in anthocyanin pigmentation in blood-fleshed peaches [97]. We employed a K-means clustering, as proposed earlier by Handhayani and Hiryanto [61], which permitted the clustering of 12,625 unique genes among the samples (F, H, and B ) into six sub-clusters with some members in Cluster 1 associated with genes from the MYB and bHLH TFs. The members in the six sub-clusters might be exploited for further downstream analyses to unravel the regulatory mechanisms of flower color formation in D. nestor. 

### 4.3. SSR Markers Discovered for Practical D. nestor Breeding

Transcriptome data provide fast and cost-effective development of molecular markers for practical plant breeding [99,100]. Specifically, SSR markers are useful tools for genomic studies and plant breeding. We detected a total of 41,286 SSR markers and developed 41,285 primers for these markers (Figure 3, Additional File 2: Table S1). It is expected that these molecular markers would facilitate genotyping and marker-assisted breeding in D. nestor and other related species.

## 5. Conclusions

We performed transcriptome analyses to study the molecular mechanisms that regulate flower color development in D. nestor. We showed that flower coloration in D. nestor is largely controlled by anthocyanins biosynthetic genes. The structural genes and co-expressed TFs reported in this study would serve as useful genetic resources for further functional characterization and molecular breeding programs in D. nestor. 

## Figures and Tables

**Table 1:** Overview of the transcriptome sequencing dataset and quality check.

Sample ^a^	Raw Reads	Clean Reads ^b^	Clean Base (G)	Q30 (%)	GC Content (%)
F1	53327576	52337746 (98.14%)	7.85	93.58	47.24
F2	52995426	49011814 (92.48%)	7.35	94.70	47.50
F3	47190160	46301832 (98.12%)	6.95	93.43	46.36
Average F	50047108	47538849 (96.25%)	7.13	94.32	47.72
H1	53098924	49694824 (93.59%)	7.45	94.00	47.98
H2	51764262	49406926 (95.45%)	7.41	94.53	47.91
H3	45278138	43514798 (96.11%)	6.53	94.42	47.26
Average H	50047108	47538849 (94.98%)	7.03	94.07	47.96
B1	52422668	50153474 (95.67%)	7.5248	94.30	47.81
B2	50331532	47943212 (95.25%)	7.19	94.17	47.74
B3	50758962	49698100 (97.91%)	6.39	93.73	48.32
Average P	51171054	48879555 (95.52%)	7.38	93.90	47.03

^a^ F = flower bud stage; H = half blooming stage; B = full blooming stage. ^b^ those in parenthesis represent ratio of clean reads relative to raw reads expressed in percentage.

**Table 2:** Twenty most abundant transcription factors among the differentially expressed genes.

TF ^a^	F_vs_H			F_vs_B			H_vs_B		
	Total ^b^	Up ^c^	Down ^d^	Total	Up	Down	Total	Up	Down
AP2/ERF	44	40	4	59	37	22	16	1	15
AUX/IAA	10	6	4	13	3	10	8	-	8
B3	29	20	6	29	13	16	3	-	3
bHLH	56	42	24	38	9	29	8	2	6
bZIP	19	12	7	24	17	7	2	-	2
C2C2	30	8	22	37	5	32	4	1	3
C2H2	25	17	8	14	14	-	1	-	1
C3H	26	20	6	23	17	6	3	2	1
GARP	21	8	13	17	7	10	-	-	-
GNAT	8	2	6	2	1	1	-	-	-
GRAS	14	12	2	12	7	5	2	1	1
HB	31	17	14	39	25	14	6	2	4
MADS	11	5	6	9	5	4	1	-	1
MYB	44	17	27	35	13	22	7	-	7
NAC	33	30	3	25	22	3	6	2	4
PHD	8	4	4	10	1	9	-	-	-
PLATZ	11	11	-	12	12	-	5	5	-
Tify	16	5	11	11	-	11	6	-	6
Trihelix	6	3	3	9	6	3	3	3	-
WRKY	37	32	5	24	18	6	13	6	7

^a^ Transcription factor. ^b^ Total of differentially expressed genes (DEGs). ^c^ upregulated. ^d^ downregulated. F = flower bud stage; H = half blooming stage; and B = full blooming stage.

## Data Availability

The datasets used or analyzed during the current study are available from the corresponding author upon reasonable request. The transcriptome raw reads were deposited in the Sequence Reads Archive (SRA) of the National Center for Biotechnology Information (NCBI), under the BioProject number PRJNA680853. The transcriptome raw reads have been deposited in the Sequence Reads Archive of the National Center for Biotechnology Information under the BioProject number PRJNA680853.

## Supplementary Materials

The following are available online at https://www.mdpi.com/2075-1729/11/2/113/s1, Figure S1. Distribution of transcript lengths and unigenes (n = total number). Figure S2. Relative expression of selected differentially expressed genes for quantitative polymerase chain reaction (qRT-PCR), among the three samples (F = flower bud stage; H = half blooming stage; B = full blooming stage). The error bar represents standard error of means. The correlation plot was constructed using qRT-PCR and RNA-seq data. *,**,*** represent significant difference between F samples and H / B samples at *p* < 0.05, 0.01, 0.001, respectively. Table S1. Primers for specific simple sequence repeats detected in each unigene (designed usingPrimer3 software). Table S2a. Differentially expressed genes detected in F_vs_H and their functional annotations. Table S2b. Differentially expressed genes detected in F_vs_B and their functional annotations. Table S2c. Differentially expressed genes detected in H_vs_B and their functional annotations. Table S3. Predominant pathways detected from Kyoto Encyclopedia of Genes and Genomes database with differentially expressed genes mutually detected among the three pairwise groups. Table S4a. Transcription factors identified among the differentially expressed genes in F_vs_H. Table S4b. Transcription factors identified among the differentially expressed genes in F_vs_B. Table S4c. Transcription factors identified among the differentially expressed genes in H_vs_B. Table S5. K-means clustering analysis based on the FPKM of 12625 unique genes among the samples (F, H and B). Table S6. Primers of selected genes used for the qRT-PCR.

## Abbreviations

COG	Clusters of orthologous groups of proteins
DEGs	Differentially expressed genes
FPKM	Fragments per kilobase of transcript per million fragments mapped
KEGG	Kyoto Encyclopedia of Genes and Genomes
KOG	Eukaryotic Clusters of Orthologous Groups
MISA	MIcroSAtellite identification Single nucleotide polymorphisms
Nr	Non-redundant protein sequence database
ORF	Open reading frame
SSR	Simple sequence repeats
STEM	Short time-series expression miner
TFs	Transcription factors
TrEMBL	Translated European molecular biology laboratory
