# Supplementary material for: Transcriptomic Analysis of the Anthocyanin Biosynthetic Pathway Reveals the Molecular Mechanism Associated with Purple Color Formation in Dendrobium Nestor
Source: Life (Basel). 2021 Feb 2;11(2):113. doi: 10.3390/life11020113 (PMC7912934; doi:10.3390/life11020113)
Supplement: Supplementary file 1 [file life-11-00113-s001.zip › Additional file 1 Fig S1.docx]

**Additional file 1 Fig S1** Distribution of length in transcripts and unigenes (n= total number)
