# Supplementary material for: Transcriptomic Analysis of the Anthocyanin Biosynthetic Pathway Reveals the Molecular Mechanism Associated with Purple Color Formation in Dendrobium Nestor
Source: Life (Basel). 2021 Feb 2;11(2):113. doi: 10.3390/life11020113 (PMC7912934; doi:10.3390/life11020113)
Supplement: Supplementary file 1 [file life-11-00113-s001.zip › Additional file 4 Fig S2.docx]

**Additional file 4 Fig S2.** Relative expression of selected differentially expressed genes (DEGs) for quantitative polymerase chain reaction (qRT-PCR) among the three samples (F = flower bud stage; H = half blooming stage; B = full blooming stage). The error bar represents standard error of means. The correlation plot was constructed using qRT-PCR and RNA-seq data. *,**,*** represent significant difference between F samples and H / B samples at p < 0.05, 0.01, 0.001, respectively.

B

H

F
